# Prevention of adhesions in gynaecological surgery: the 2012 European field guideline

**DOI:** 10.1007/s10397-012-0764-2

**Published:** 2012-08-24

**Authors:** Rudy Leon De Wilde, Hans Brölmann, Philippe Robert Koninckx, Per Lundorff, Adrian M. Lower, Arnaud Wattiez, Michal Mara, Markus Wallwiener, The Anti-Adhesions in Gynecology Expert Panel (ANGEL)

**Affiliations:** 1 Pius-Hospital Frauenklinik, Oldenburg, Germany; 2 VU University, Amsterdam, The Netherlands; 3 University Hospital Gasthuisberg, Katholieke Universiteit, Leuven, Belgium; 4 Viborg Hospital, Viborg, Denmark; 5 The Womens’ Health Partnership Ltd, London, UK; 6 Hôpital de Hautepierre, Strasbourg, France; 7 Charles University, Prague, Czech Republic; 8 Department of Obstetrics and Gynaecology, University of Heidelberg, Vosstr. 9, Heidelberg, 69115 Germany

**Keywords:** Adhesions, Adhesiolysis, Adhesion prevention, Treatment guideline, Anti-adhesion agents

## Abstract

Postoperative adhesions have become the most common complication of open or laparoscopic abdominal surgery and a source of major concern because of their potentially dramatic consequences. The proposed guideline is the beginning of a major campaign to enhance the awareness of adhesions and to provide surgeons with a reference guide to adhesion prevention adapted to the conditions of their daily practice. The risk of postoperative adhesions should be systematically discussed with any patient scheduled for open or laparoscopic abdominal surgery prior to obtaining her informed consent. Surgeons should adopt a routine adhesion reduction strategy with good surgical technique. Anti-adhesion agents are an additional option, especially in procedures with a high risk of adhesion formation, such as ovarian, endometriosis and tubal surgery and myomectomy. We conclude that good surgical practice is paramount to reduce adhesion formation and that anti-adhesion agents may contribute to adhesion prevention in certain cases.

Postoperative adhesions—fibrous connections developing between tissues and organs as a sequel to surgical trauma—have become the commonest complication of open or laparoscopic abdominal surgery and a source of major concern because of their potentially dramatic consequences.

Only a few specialists are aware of the extent of the adhesions problem. Adhesions are a complication of surgery and the problems they are causing can be severe. The lack of awareness about adhesions and adhesion-related disease makes many doctors unable to take care, insurance companies unwilling to pay, and patients left with their complaints.

Regarding the fact that nearly every abdominal surgery causes adhesions, bowel obstructions due to the adhesions can cause death and many patients have persistent pain, dyspareunia, infertility or bowel complaints after operations, it is amazing that there is such a lack of interest and scientific investigations.

Adhesiolysis, the most common treatment of postoperative adhesions, is too often followed by adhesion reformation. To ensure that their patients receive the best standard of care and avoid adhesion-related litigation claims, surgeons should routinely adopt effective measures to prevent postoperative adhesions.

Several consensus statements on adhesion prevention give similar recommendations based on available evidence [1–5]. However, the format of these academic documents may be less practical for the busy gynaecological surgeon.

The proposed guideline is the beginning of a major concept and work in order to enhance the awareness of adhesions in general, make the scientific research grow and at the end reduce the adhesion-related disease in our patients.

This “field guideline” written by a panel of European Experts aims to provide surgeons with a quick reference guide to adhesion prevention adapted to the conditions of their daily practice. Postoperative adhesions—fibrous connections developing between tissues and organs as a sequel to surgical trauma—have become the commonest complication of open or laparoscopic abdominal surgery and a source of major concern because of their potentially dramatic consequences.

Only a few specialists are aware of the extent of the adhesions problem. Adhesions are a complication of surgery and the problems they are causing can be severe. The lack of awareness about adhesions and adhesion related disease makes many doctors unable to take care, insurance companies unwilling to pay, and patients left with their complaints [6, 7].

Regarding the fact that nearly every abdominal surgery causes adhesions, bowel obstructions due to the adhesions can cause death and many patients have persistent pain, dyspareunia, infertility or bowel complaints after operations, it is amazing that there is such a lack of interest and scientific investigations.

Adhesiolysis, the most common treatment of postoperative adhesions, is too often followed by adhesion reformation. To ensure that their patients receive the best standard of care and avoid adhesion-related litigation claims, surgeons should routinely adopt effective measures to prevent postoperative adhesions.

Several consensus statements on adhesion prevention give similar recommendations based on available evidence [1–5]. However, the format of these academic documents may be less practical for the busy gynaecological surgeon.

The proposed guideline is the beginning of a major concept and work in order to enhance the awareness of adhesions in general, make the scientific research grow and at the end reduce the adhesion related disease in our patients.

This “field guideline” written by a panel of European Experts aims to provide surgeons with a quick reference guide to adhesion prevention adapted to the conditions of their daily practice.

## What you should know about postoperative adhesions and their consequences

Adhesions have become the most frequent complications of abdominal surgery—93 % of patients undergoing any abdominal/pelvic surgery are affected [5]—and an important source of postoperative problems

- The overall risk of adhesion-related readmission following either laparoscopic or open surgery is comparable [8]
- Over one third of patients who undergo extensive open surgery seem to be readmitted with adhesion-related complications within 10 years [9]
- Adhesions are involved in 56 % of reintervention complications [10]
- Seventy-four percent of cases of bowel obstruction are due to post-surgical adhesions [11]
- Adhesions are associated with a marked risk of enterotomy jeopardising 19 % and 10–25 % of patients undergoing open and laparoscopic surgery, respectively [12, 13]
- Adhesions are responsible for 20–40 % of secondary infertility cases in women [14, 15]

In addition, adhesions generate a high number of reinterventions, increase hospital stays, extend reintervention times and can make it impossible to apply minimally invasive surgery. Last but not least, managing adhesions and their related complications impose an enormous economic burden. In the UK, the cost of adhesion-related readmissions was estimated at £24.2 and £95.2 million at 2 and 5 years after surgery, respectively [16]

## The six basic rules of postoperative adhesion prevention in gynaecological surgery [2]

1. The risk of postoperative adhesions should be systematically discussed with any patient scheduled for open or laparoscopic abdominal surgery prior to obtaining his/her informed consent
2. Surgeons need to act to reduce postoperative adhesions in order to fulfill their duty of care towards patients undergoing abdominal surgery
3. Surgeons should adopt a routine adhesion reduction strategy at least for patients undergoing high-risk surgery, including:
  - Ovarian surgery
  - Endometriosis surgery
  - Tubal surgery
  - Myomectomy
  - Adhesiolysis
4. Good surgical technique is fundamental to any adhesion reduction strategy
  - Carefully handle tissue with field enhancement (magnification) techniques
  - Focus on planned surgery and, if any secondary pathology is identified, question the risk: benefit ratio of surgical treatment before proceeding
  - Perform diligent haemostasis and ensure diligent use of cautery
  - Reduce cautery time and frequency and aspirate aerosolised tissue following cautery
  - Excise tissue—reduce fulguration
  - Reduce duration of surgery
  - Reduce pressure and duration of pneumoperitoneum in laparoscopic surgery
  - Reduce risk of infection
  - Reduce drying of tissues
  - Use frequent irrigation and aspiration in laparoscopic and laparotomic surgery when needed
  - Limit use of sutures and choose fine non-reactive sutures
  - Avoid foreign bodies when possible—such as materials with loose fibres
  - Avoid non-peritonised implants and meshes
  - Minimal use of dry towels or sponges in laparotomy
  - Use starch- and latex-free gloves in laparotomy
5. Surgeons should consider the use of adhesion reduction agents as part of the adhesion reduction strategy
  - Give special consideration to agents with data supporting safety in routine surgery and efficacy in adhesion prevention
  - Practicality, ease of use, and cost of agents should influence their selection for routine practice
6. Good medical practice implies that any serious or frequently occurring risks be discussed before obtaining the patient’s informed consent prior to surgery

For women undergoing gynaecological surgery, and particularly those undergoing tubal and ovarian surgery procedures, who wish to conceive, the implementation of good surgical practice, together with the adoption of adhesion-reduction agents, is paramount to reduce adhesion formation. As all healthcare providers, surgeons have the duty to protect patients by providing the best standards of care—this includes taking steps to reduce adhesion formation.
